# Host-microbiome interactions and enteric pathogen infections—from early-life to long-term impacts on gut-brain communication

**DOI:** 10.1093/jambio/lxag237

**Published:** 2026-09-15

**Authors:** Mélanie G Gareau, Jungjae Park, Kim E Barrett

**Affiliations:** Department of Anatomy, Physiology, and Cell Biology, School of Veterinary Medicine, University of California, Davis, Davis, CA 95616, United States; Department of Anatomy, Physiology, and Cell Biology, School of Veterinary Medicine, University of California, Davis, Davis, CA 95616, United States; Department of Physiology and Membrane Biology, School of Medicine, University of California, Davis, Sacramento, CA 95817, United States

**Keywords:** Microbiome, intestinal pathogen, gut-brain axis, host-microbe interations

## Abstract

Interactions of the host with the microbes that constitute the intestinal microbiota are important for maintaining health and well-being within the gastrointestinal tract and beyond. Disruptions to these interactions, such as those that follow enteric pathogen infection or administration of antibiotics, can lead to persistent pathophysiology, particularly if disruptions occur during early development in neonatal life. Here, we highlight the impacts of altered host-microbe interactions following insults across the life span and discuss emerging mechanisms of action promoting these disruptions (see Graphical Abstract). We also identify potential therapeutics to restore these important pathways and their mechanisms of action.

## Introduction

Microbial colonization of the gut and the establishment of intestinal barrier function occur simultaneously during early neonatal development. Intestinal epithelial cells (IEC) function in concert with gut-resident commensal bacteria (the intestinal microbiota) as well as the combination of these microbes, their genetic potential and metabolic products, and environmental factors (the intestinal microbiome) in a mutualistic relationship that develops over time. This interaction occurs despite the fact that IEC and gut microbes typically are not in direct contact with each other, due to the presence of mucus layers and epithelial cell junctions, which serve as intermediate structures critical to maintaining host-microbe separation while allowing vital communication. Similarly, the developing microbiome instructs appropriate development of the enteric nervous and mucosal immune systems, which also contribute to a balanced relationship between the host and its associated microbes (Macpherson et al. 2023). Disruption of this homeostasis, following enteric pathogen infection (bacterial, viral, or parasitic), or administration of antibiotics, can lead to a breakdown in the mucus layer, alterations in the composition of the gut microbiome, and impacts on signaling within IEC, nerves, and immune cells, resulting in impaired host-microbe interactions. When this disruption occurs during the critical window during which these interactions develop, it can lead to lifelong impairments. Delineating the exact processes involved in the establishment of host-microbe interactions in early life, the role of the multiple factors at play, and how they can be restored following disruption are key components to better understanding the acute and chronic impacts of enteric pathogen exposure on the host. Similarly, at the other extreme of life, typical changes occurring to the microbiome with aging also have implications for host susceptibility to gastrointestinal infections and gut-brain communication.

## Programming of gut-brain communication across the lifespan

### Early life

The microbiota, gut, and brain communicate bidirectionally to regulate a multitude of homeostatic processes (Cryan et al. 2019). Therefore, disrupting colonization by the gut microbiota in early life can have detrimental impacts that persist across the lifespan, and impact the whole body as development and maturation continue to occur over time. The neonatal, pre-weaning period is critical in the development of tolerance to gut bacteria and the establishment of a mutualistic relationship between the host and its microbes (Knoop et al. 2017). This is characterized as the window of time during which the “weaning reaction” takes place, whereby the gut microbiome expands in extent and complexity, imprinting immunoregulatory responses on the host during the dietary transition period from milk to solid food (Al Nabhani et al. 2019). In humans, the bacterial components of the infant microbiome shift from Bifidobacteria to Bacteroidota and Firmicutes in breast-fed babies over time, yielding a more diverse and complex stable ecosystem (Wu et al. 2025). In mice, the gut microbiome shifts from dominance by Lactobacillales and Proteobacteria to Clostridia and *Bacteroides* during the change from predominantly milk-based nutrition to complementary solid foods (Al Nabhani et al. 2019). In the 2 weeks post-weaning, stability of the microbiota is established, with changes in the relative abundance of bacterial populations occurring during this period without a change in taxa, and remaining stable for months (Schloss et al. 2012). Interfering with the weaning reaction in mice was found to cause pathological immune imprinting and increased the susceptibility to disease, including colitis, allergy, and cancer, in later life (Al Nabhani et al. 2019). The subsequent post-weaning period is associated with increased stability of the gut microbiome and reduced plasticity, along with the establishment of appropriate host responses to insults and injury that continue to evolve throughout life.

The lining of the GI tract is composed of columnar IEC joined together via tight junction proteins that are critical for maintaining the epithelial barrier and limiting access of luminal contents to the underlying lamina propria. During the weaning transition period, the GI tract matures gradually as it adapts to evolving feeding patterns. Prolactin in milk regulates IEC maturation, including modulation of cell proliferation in neonatal mice (Dena-Beltran et al. 2025). Human milk oligosaccharides (HMO) also help promote proliferation of IEC and their differentiation into goblet cells, Paneth cells, and enteroendocrine cells (Liao et al. 2025). HMOs are also known to promote colonization with beneficial microbes, particularly *Bifidobacterium infantis*. Metabolites produced by *B. infantis*, including indole-3-lactic acid, can significantly attenuate inflammatory responses in IEC *in vitro* (Ehrlich et al. 2020). Synergistic effects were found when combining HMOs with probiotics, with 3′-sialyllactose plus *Bifidobacterium infantis* supplementation ameliorating colonic injury, increasing intestinal barrier function, and increasing tight junction protein expression in a mouse model of chemically induced colitis (Yang et al. 2025). While distinct HMOs modulate host inflammation and barrier function, they activate different signaling pathways, as determined by transcriptome analysis, highlighting important structure-function relationships (Wu et al. 2022). Further, parasitic pathogens commonly occur in children in the developing world, and can cause significant diarrheal disease. Infection with *Cryptosporidium* and *Giardia* can disrupt IEC tight junctions both in mice and in human cell lines *in vitro*, impairing the mucosal barrier and leading to infection (Liu et al. 2018, Luo et al. 2024). *Giardia* also releases cysteine proteases that serve as virulence factors and can degrade multiple chemokines, impairing the host immune response to infection (Liu et al. 2018). Therefore, the transition during weaning represents an important period of development of host-microbe interactions and establishment of the mucosal barrier.

Within the lamina propria, enteric neurons play a critical role in modulating both IEC function and overall GI physiology, and even promoting gut-brain communication. Early exposure of enteric neurons to microbial products, during the window of barrier immaturity, induces expression of the transcription factor, aryl hydrocarbon receptor (AHR). AHR in turn subsequently allows the enteric nervous system to respond to microbial products with the development of peristaltic and other motility patterns (Obata et al. 2020). AHR activation is also known to enhance IEC barrier function as well as the function of mucosal immunocytes. In turn, these actions contribute to control of the composition of the microbiome. Intestinal parasites, including *Cryptosporidium* and *Giardia*, can also impact enteric neurons and contribute to post-infectious GI pathology in mice, although the mechanisms for this remain poorly understood (Halliez and Buret 2015). All these important early life interactions help shape the microbiome, IEC development, GI physiology, and the subsequent establishment of beneficial host-microbe interactions, and help to protect against infection with enteric pathogens.

### Pre-weaning period

During the pre-weaning period, host-microbe signaling is initiated and continues to develop as the intestinal microbiome matures, leading to the emergence of multiple intestinal defense mechanisms. The pre-weaning diet is a significant contributor to microbiome maturation, with formula feeding versus breastfeeding having a significant impact on intestinal enterotypes during the transition period in humans (Sawhney et al. 2025). Maternal IgG, transferred to offspring during lactation in the first weeks of life, helps to establish mucosal immune responses and maintains host-microbiome adaptive immunity for multiple weeks, extending post-weaning in mice (Shenoy et al. 2025). Exposure to enteric pathogens during this developmental period can disrupt this process. For example, neonatal infection can detrimentally impact IEC maturation, as demonstrated following *Salmonella* infection in mice. Accelerated maturation of IEC following infection causes premature appearance of antimicrobial, metabolic, developmental, and regenerative features in the GI tract (Schlosser et al. 2025). Infection-induced innate immune signaling triggers changes in gene expression, disrupting the maturation of the gut (Schlosser et al. 2025). Cytokine stimulation of neonatal intestinal epithelial stem cells leads to infection-induced enterocyte reprogramming, also potentially contributing to altering the neonatal-adult transition period (Schlosser et al. 2025). This effect was restricted to neonatal mice, highlighting the unique features of early development that make it more susceptible to long-lasting impairments following disruption.

The tissue-adherent inner mucus layer, a key player in the maintenance of mucosal barrier function that is impenetrable by most microbes, develops during the pre-weaning period. Microbial colonization plays an important role in driving postnatal goblet cell maturation, and establishment of the mature mucus layer (Johansson et al. 2025). The enteric nervous system also continues to develop and mature during the pre-weaning period in mice, with the gut microbiome playing an important role in this development (Obata et al. 2020). This is highlighted in the setting of neonatal antibiotic administration, which can impact neurochemistry detrimentally in both the myenteric and submucosal plexus and cause delayed GI transit times that persist into adulthood (Poon et al. 2022). These findings suggest that maturation of these mucosal defense mechanisms during early life can contribute to the establishment of multiple facets of GI physiology.

Concurrent with microbial colonization, postnatal neurodevelopment in the gut and brain also occurs, with significant neurogenesis in both compartments. This overlap suggests that dysbiosis of the gut microbiome during early life can impact these processes and contribute to impairments in neurons both in the gut and within the brain. High rates of neurogenesis occurring during this early period suggest that an impairment in this process may potentially impact behavior, and can even persist later in life. Neonatal infection, including with enteropathogenic *Escherichia coli* (EPEC), causes altered colonization of the gut microbiota and GI pathophysiology that remains into adulthood, well after clearance of the pathogen (Hennessey et al. 2021). This is accompanied by cognitive deficits in the presence of neuroinflammation and altered neurogenesis in the hippocampus of adult mice (Hennessey et al. 2021). These studies highlight a long-lasting gut-brain deficit following neonatal enteric bacterial infection.

Taken together, these studies highlight the important role of the neonatal period for establishment of host-microbe interactions and the potential long-term impacts of any deficits in this system both within the gut and on extra-intestinal communication pathways. Future mechanistic studies identifying the specific pathways involved in regulating the transition during weaning, including physiological, immunological, and neural, are key to understanding how to restore homeostasis following neonatal infection, particularly in humans.

### Post-weaning period

The post-weaning transition period is particularly important for maturation of the gut microbiome, the intestinal epithelium, and the mucosal immune system. During this time, the gut epithelium begins to exhibit more robust barrier properties, maternal immunoglobulins decrease in quantities, and the microbiome increases in stability (Westrom et al. 2020). If this transition is disrupted, for example by transient administration of antibiotics starting at weaning, it can lead to long-lasting dysbiosis of the gut microbiome coupled with the presence of altered behavior, including alterations in anxiety-like behavior in mouse models (Lach et al. 2020). This should be contrasted with the short-term effects on the composition of the microbiome and downstream behavioral states that are typically seen when antibiotics are initiated in adulthood. The impact of timing extends to impacts on the enteric nervous system, with post-weaning vancomycin administration increasing transmission and responsiveness in both myenteric and submucosal neurons, impacting inhibitory motor pathways and causing slower motility patterns in the mouse colon (Hung et al. 2020). Arresting the development of the microbiome at weaning also stunts immune system development and increases susceptibility to enteric bacterial infections, including *Salmonella* (Lubin et al. 2023). Similarly, induction of colonic inflammation at weaning in mice, using the colitogenic agent dextran-sodium sulfate (DSS), caused persistent gut dysbiosis, GI inflammation, behavioral impairments, and neuroinflammation in adulthood (Salvo et al. 2020). Taken together, the post-weaning transition period is important for the maturation of multiple aspects of host-microbe interactions and extra-intestinal development, with disruption during this time period associated with long-lasting deficits. While post-weaning toddlers are known to be highly susceptible to enteric pathogens (Kotloff et al. 2013), in-depth human clinical studies are missing and would be valuable in determining the long-term risks imposed by enteric pathogens during this period in childhood.

### Aging

At the other end of the lifespan, a group known also to be especially susceptible to the adverse effects of infection with enteric pathogens is the elderly. Not only are older humans more susceptible to specific enteric infections, the consequences of these infections may be more profound and include diarrheal symptoms, dehydration, and cognitive dysfunction as well as an increased risk of mortality even following brief diarrheal illnesses (Dawod and Crawford 2021). While the typical GI dysfunction in the elderly is constipation, perhaps secondary to diminished intake of dietary fiber, the microbiome evolves over the lifespan and is less diverse in old age than in early adulthood, which contributes to a decrease in SCFA production (Ragonnaud and Biragyn 2021). This less diverse microbiome may also lead to diminished resistance to pathogen colonization, but also altered microbiome-gut-brain signaling via specific bacterial metabolites. The dysbiosis seen with aging also correlates with numerous non-GI diseases and also with frailty (Lim and Nam 2023); those individuals who remain fit and healthy at advanced age display microbiomes that are comparable to those of younger adults. The dysbiotic microbiome associated with aging has altered functional capabilities and has been implicated in decreases in the efficacy of the blood-brain barrier and thus to neurodegenerative diseases (Olejnik et al. 2025). This imbalance also contributes to “inflammaging,” where persistent low-grade inflammation contributes to the progression of aging, including frailty—nutrition, including caloric restriction, and the composition of the gut microbiome (Di Giosia et al. 2022) are important components of healthy aging and protection against enteric pathogens in both mice and humans.

Recurrent overgrowth of *Clostridium difficile* in the gut, often secondary to antibiotic administration, is a particular problem in elderly patients and especially those that are hospitalized or living in other institutionalized settings. The declining ability of the dysbiotic microbiota in these patients to compete with *C. difficile* is illustrated by the efficacy of fecal microbiota transplant (FMT) from healthy individuals to resolve both *C. difficile* overgrowth and its associated symptoms (Vemuri et al. 2017). Taken together, these findings highlight the importance of a diverse gut microbiome in older age, and suggests that improving ways to maintain high diversity could be a critical component of overall healthy aging.

## Diarrheal pathogens and disruption of host-microbe interactions

### Host responses

Diarrheal disease caused by bacterial, viral, and parasitic pathogens is the second leading cause of morbidity and mortality worldwide. The pathogenesis of these conditions includes, at least in part, an inhibition of absorptive epithelial solute transporters, disrupting normal water and ion flows, and leading to increased net fluid and electrolyte secretion and subsequently causing clinical diarrhea (Das et al. 2018). Active chloride secretion may also be induced by certain toxigenic pathogens. Further, following colonization, pathogen-associated virulence factors are produced, causing activation of mucosal immune responses and leading to inflammation, which may in turn worsen diarrhea. While these host responses are typically acute in nature, and sequalae can typically be treated effectively with oral rehydration solution for many enteric pathogens, they are also strongly associated with long-term, persistent deficits (including in cognition) in a subset of individuals, particularly the young and elderly, who are more susceptible to enteric infections (Scharf et al. 2014, Fernandez-Cotarelo et al. 2023).

Pathogen-induced diarrhea is an important host response to infection, which helps clear pathogens out of the gut and limit further colonization (Fig. 1). The mechanisms of pathogen-induced diarrhea vary and include altering fluid and electrolyte transport by interfering with apical transporters, damage to IECs causing cell death, and/or initiation of inflammation caused by and causing barrier dysfunction, establishing a vicious cycle. For example, pathogen-induced increased production of the regulatory cytokine IL-22 in mice upregulates expression of the pore-forming claudin-2, driving increasing permeability and promoting diarrhea via enhanced water efflux into the lumen (so-called “leak-flux” diarrhea) (Tsai et al. 2017). *Citrobacter rodentium*-infection of mice caused enhanced disease in claudin-2 deficient mice, whereas disease severity was reduced in claudin-2 transgenic mice highlighting this critical role of claudin-2 (Tsai et al. 2017). This was in part due to the ability of claudin-2 to promote pathogen clearance and limit mucosal immune activation via IL-22 (Tsai et al. 2017). Similarly, the DRA (down-regulated in adenoma) chloride/bicarbonate exchanger was targeted following inoculation of neonatal mice with inactivated rotavirus via increased interferon λ production by IECs, resulting in decreased water absorption and diarrhea (Hou et al. 2025). In *C. difficile* infection of mice, a decrease in DRA was also found, coupled with impairment of the cystic fibrosis transmembrane conductance regulator (CFTR) in the distal colon, whereas the calcium-activated chloride channel (CaCC) was severely impaired in the cecum of such animals (Peritore-Galve et al. 2023). Similarly, blocking of CFTR with a small molecule inhibitor prevented diarrhea in a mouse model of *Vibrio cholera* infection (Rivera-Chavez et al. 2022), highlighting an important role for active chloride secretion in mediating disease following certain enteric infections. Following infection with enterotoxigenic *E. coli* (ETEC) in mice, carcinoembryonic cell adhesion molecules (CECAM) on IEC are exploited by the pathogen to deliver its heat-labile toxin and cause diarrheal disease (Sheikh et al. 2024). Lastly, bone morphogenic protein (BMP), important for tissue repair, can be activated by *V. cholerae* to block epithelial cell proliferation in Drosophila and zebrafish models, highlighting an evolutionarily conserved pathway in response to enteric infection (Xu and Foley 2024). In sum, all of these pathways can be activated alone or in combination to drive diarrheal responses to pathogenic infection.

**Figure 1:**
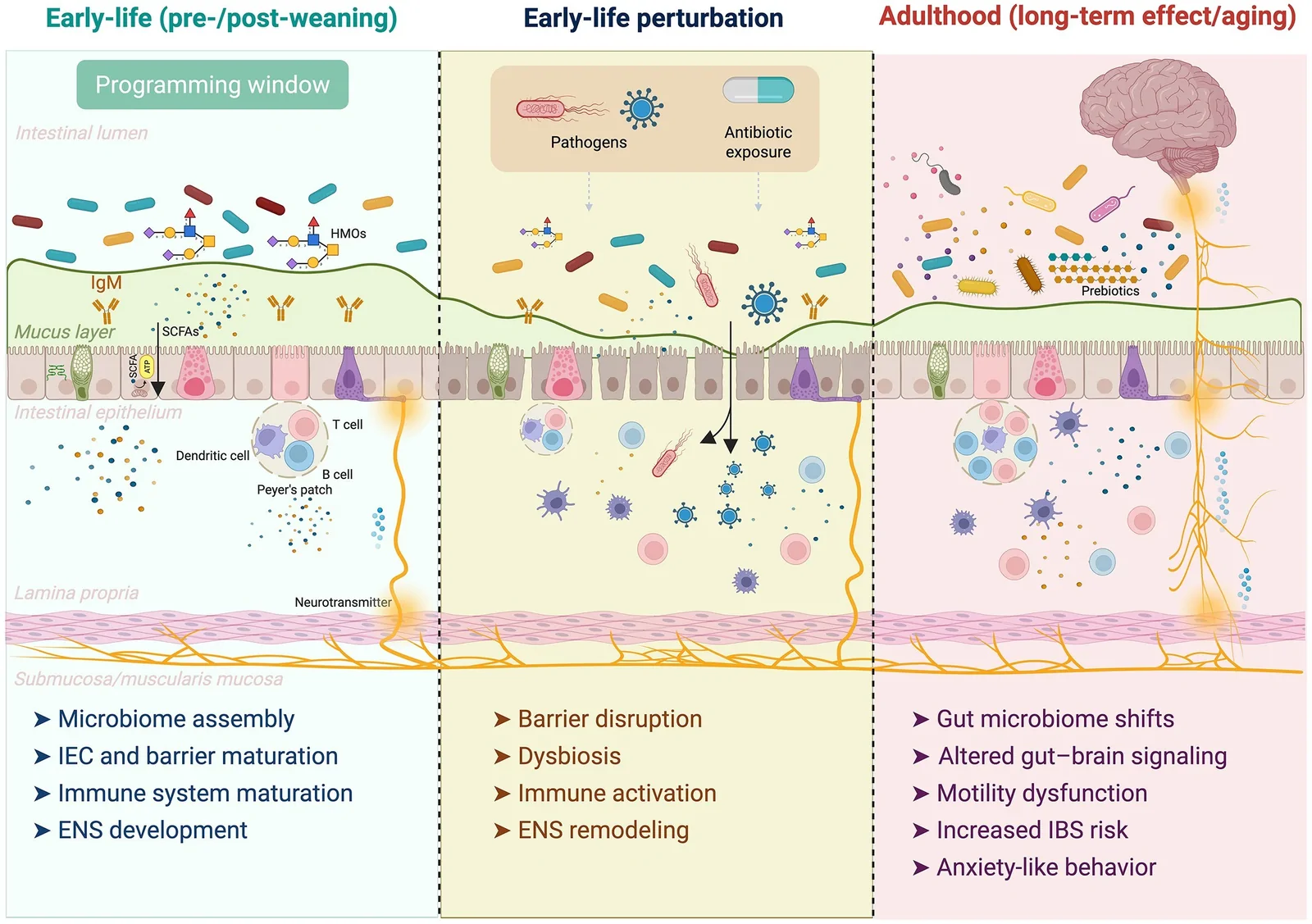
During early life, encompassing the pre- and post-weaning period, the gut undergoes rapid microbiome assembly, mucosal barrier maturation, and development of intestinal epithelial, immune, and enteric nervous system (ENS) networks, representing a critical programming window. Enteric infections or antibiotic exposure during this period disrupt epithelial barrier integrity and microbiota composition, leading to dysbiosis, immune activation, and ENS remodeling. These early-life perturbations imprint persistent alterations in gut–brain signalling that extend into adulthood, manifesting as changes in gastrointestinal motility, increased risk ofpost-infectious functional bowel disorders, and anxiety-like behavior. Together, the graphical abstract highlights how transient insults during early development can drive long-term physiological and neurobehavioral outcomes through sustained remodeling of the microbiota–gut–brain axis.

In addition to IEC responding directly to pathogens, they can also produce molecules that interface with pathogens within the lumen. For example, defensins are Paneth cell-derived molecules that protect the host from bacterial pathogens by helping keep the base of the crypt sterile. *Shigella* can exploit these innate immune peptides, turning them into virulence factors and co-opting them to enhance adhesion and invasion of epithelial cells (Xu et al. 2018). For example, *Shigella* can exploit human enteric α-defensin 5 to trigger the formation of filipodial extensions on colonic epithelial cell membranes and thereby enhance capture of the bacteria and their subsequent invasion of host epithelial cells both *in vitro* and in an *in vivo* mouse model (Xu et al. 2025). IECs can also initiate innate immunes responses directly in response to pathogen infection, such as in rotavirus infection, where Ifnλ is produced by IEC as determined using an *in vitro* organoid culture system (Hou et al. 2025). Similarly, *Cryptosporidium* infection is prevented in mice lacking Ifnar in their IEC, suggesting IEC type 1 IFN production is crucial for host antiparasitic responses (Deng et al. 2023). Goblet cells can also serve as important regulators of viral infection, with mucin synthesis inhibiting IEC infection by porcine epidemic diarrhea virus (PEDV) *in vitro* and, conversely, PEDV able to suppress mucin secretion by porcine intestine *in vivo* to facilitate infection (Wang et al. 2025).

Infection with enteric pathogens elicits numerous host responses, initiated in large part by IEC and their secreted molecules. This process, however, is heavily influenced by multiple factors, including changes to the luminal environment, the availability of vitamins and minerals, oxygenation, and the production of neurotransmitters as examples. All of these host factors also depend heavily on the composition of the gut microbiome, and work together to respond to enteric pathogens.

### Luminal factors

The luminal environment is important for maintaining homeostasis and providing resistance against invading pathogens, including changes in pH, flow, and motility, as well as the composition of the resident commensal microbiome (Sommer et al. 2017). The gut microbiome can provide protection against enteric pathogens through multiple pathways, including competition for nutrients, production of inhibitory molecules, or priming innate immune host responses.

The pH within the intestinal lumen varies along the length of the GI tract and is crucial for maintaining digestive processes across the different regions. In addition, it plays an important role with respect to pathogen colonization by triggering microbial cues as organisms migrate along the length of the gut. Homeostatic changes in environmental pH along the gut can elicit virulence gene expression to promote survival and growth of pathogens within the host, allowing effective infection within specific regions (Woodward et al. 2024). For example, EPEC typically infects and colonizes the small intestine, whereas enterohemorrhagic *E. coli* (EHEC) is typically found within the large intestine. Luminal flow and peristaltic contractions can also contribute to infectivity of bacterial pathogens, as demonstrated using 3D organ-on-chip technologies (Grassart et al. 2019).

Bacterial metabolites within the gut lumen typically provide a healthy environment for both the host and the commensal microbiota. Short-chain fatty acids (SCFA) are highly abundant bacterial metabolites in the lumen of the gut that (1) promote colonization by commensal organisms, (2) serve as an energy source for colonocytes, and (3) can restrict the growth of pathogens. Luminal SCFA impact colonization by enteric pathogens, with increased levels of butyrate-producing bacteria within the gut microbiome associated with delayed colonization by *C. rodentium* in mice (Osbelt et al. 2020). Conversely, depletion of beneficial SCFA, including butyrate and acetate, causes a break in colonization resistance provided by the commensal microbiome and allows colonization by bacterial pathogens such as *Salmonella* in mice (Rogers et al. 2024). *Salmonella* can also overcome the inhibitory effects of SCFA, particularly propionate, by using these compounds as a carbon source for anaerobic respiration (Shelton et al. 2022). Lastly, virulence factors produced by *Salmonella* can also induce malabsorption of dietary amino acids in the small intestine, causing a shift in the availability of nutrients within the large intestine, which allows *Salmonella* to overcome the protective role of SCFA (Radlinski et al. 2024). Therefore, the availability of particular carbon sources, the amount of luminal oxygen, and nutrient composition can serve to promote or inhibit colonization resistance to pathogens.

The availability of certain dietary nutrients within the lumen can also modulate enteric bacterial infection, including selective carbohydrates and amino acids. For example, the presence of galactose-utilizing *E. coli* strains in mice can limit colonization by *Salmonella* by consuming this critical essential nutrient necessary for the latter’s survival, effectively starving it out of the gut (Schubert et al. 2025). Amino acids within the gut lumen can also modulate colonization resistance to pathogens. In *C. rodentium* infection, the presence of a microbiome in specific pathogen-free mice limits the availability of certain essential amino acids in the lumen, preventing colonization when compared to germ-free mice infected with the pathogen. When mice were supplemented with a high-protein diet, pathogen colonization in the gut was enhanced secondary to increased availability of amino acids, but only when provided early in the infection phase, not at the peak of expansion (Caballero-Flores et al. 2020). Modifying the composition of carbohydrates and/or amino acids within the lumen through dietary means may thus serve as an effective way to limit pathogen colonization.

### Iron sequestration

Siderophores are high-affinity iron chelators produced by bacteria, including pathogens, that assist the bacteria with iron acquisition. Enterobactin, for example, is found in *E. coli* and *Salmonella* and scavenges iron, promoting colonization by these pathogens. Host-derived lipocalin-2 can sequester enterobactin, serving as a key pathway to limit pathogen infection via nutritional immunity. Commensal microbes, including *Bacteroides thetaiotamicron*, can also exploit *Salmonella*-produced enterobactin by releasing a siderophore-binding lipoprotein and thereby blocking iron acquisition by the pathogen in a mouse model of infection (Spiga et al. 2023). Similarly, siderophore-directed immunization decreased colonization and systemic dissemination of *Salmonella* in mice (Sassone-Corsi et al. 2016). Taken together, these findings highlight the important role siderophores may play in limiting enteric pathogens by exploiting mechanisms inherent in the organisms to promote their colonization and survival.

### Hypoxia

Oxygen tension changes as one moves distally along the length of the gut, with the colonic lumen considered to exist in a state of “physiological hypoxia.” As such, the availability of oxygen is an important determinant of the characteristics of the microbiome (Sommer et al. 2017, Rose et al. 2025). Systemic reductions in oxygen availability (such as those induced by repeated intermittent exposure of experimental animals to hypoxia, modeling obstructive sleep apnea) can result in dysbiosis (Lu et al. 2025). Conversely, hyperbaric oxygen treatment of mice also modulates the composition of the microbiome, reducing the presence of anaerobic bacteria and the concentration of SCFA that they are largely responsible for generating (Fachi et al. 2024).

Changes in oxygen tension can also result in variations in the susceptibility of the host to enteric pathogens, which may be mediated, at least in part, by associated changes in the composition of the microbiome. For example, in the murine hyperbaric oxygen model discussed above, the relative lack of SCFA increased infection with *C. difficile*, an outcome that was associated with diminished epithelial barrier function and a failure to activate innate lymphoid cells (Fachi et al. 2024). Importantly, these effects could be reversed by exogenous delivery of butyrate. On the other hand, Rogers et al. (2024) showed that another pathogen, *Salmonella*, exploits an increase in epithelial oxygenation, secondary to virulence-factor-induced inflammation, to metabolize simple sugars and thereby drive pathogen expansion in mice. Notably, these effects occur prior to an overt change in the composition of the commensal microbiome, but rather can be ascribed to the ability of reactive nitrogen species, generated as a part of the inflammatory response to the pathogen, to modulate the metabolic functions of the microbiome and thereby reduce SCFA production. Finally, the impact of oxygen availability on pathogen virulence may depend pivotally on the pathogen in question. Recent work in epithelial cells studied *in vitro* suggests that the hypoxic environment of the large intestine in fact may be permissive for infection with viral diarrheal pathogens, such as rotavirus, by suppressing interferon production and downstream antiviral protection (Jacobs et al. 2025).

### Neurotransmitters

Neurotransmitters are endogenous chemicals not only important for neurotransmission, but which are also known to impact enteric pathogens. While they are primarily thought of as being host-derived, many neurotransmitters are also produced by select bacterial species. 5-Hydroxytryptamine (5-HT), commonly known as serotonin, is an abundant neurotransmitter in the GI tract produced by both enteric neurons and enteroendocrine cells within the epithelium, where its production can be modulated by spore-forming bacteria within the microbiome (Yano et al. 2015). 5-HT can promote decreases in the expression of virulence genes by attaching and effacing pathogens of mice (i.e. EHEC and *C. rodentium*), by binding to a bacterial serotonin receptor (CpxA) (Kumar et al. 2020). 5-HT subsequently induces dephosphorylation of CpxA, which inactivates a key transcription factor (CpxR), critical for controlling gene expression of virulence factors within the locus of enterocyte effacement pathogenicity island. Therefore, an increase in intestinal 5-HT levels decreases bacterial loads following infection, suggesting a novel therapeutic benefit of 5-HT agonists (Kumar et al. 2020). Nitric oxide can likewise inhibit growth of *Giardia in vitro* and *in vivo*, suggesting a role for neurotransmission as a host-protective role in parasitic infection (Maloney et al. 2015).

Host immune cell-derived acetylcholine (ACh) also plays an important role in limiting disease following enteric infection. *Citrobacter rodentium* infection and associated inflammation were significantly enhanced in mice selectively deficient in choline acetyltransferase (ChAT) in T cells, highlighting a protective role (Ramirez et al. 2019). In contrast, in parasitic infection with *Entamoeba histolytica*, ACh upregulates expression of virulence factors when binding to receptors on the plasma membrane and increases disease severity, as demonstrated in a hamster model of infection (Medina-Rosales et al. 2020). Therefore, modulation of neurotransmitter levels in the GI tract may serve as an important means by which enteric infections can be controlled.

Nociceptive neurons have also been found to sense enteric pathogens and defend against infection. Transient receptor potential vanilloid 1 (TRPV1)+ neurons were found to respond to *Salmonella* infection in mice with the release of calcitonin gene-related peptide (CGRP) via a mechanism involving M cells and Peyer’s patches in the small intestine, and thereby limiting pathogen invasion (Lai et al. 2020). Similarly, *C. rodentium* colonization was significantly enhanced in TRPV1-deficient mice, with increased bacterial burden and histopathology compared to wild-type controls (Cremin et al. 2023). Together, these findings highlight an important role for sensory nociceptors in the GI tract in controlling infection by enteric pathogens. Similarly, endocannabinoids, including 3-arachindonolyl glycerol, can inhibit the induction of virulence factors in *Enterobacteriaceae* pathogens, including *C. rodentium* and *Salmonella*, in part by antagonizing bacterial quorum-sensing receptors that promote the activation of bacterial secretion systems necessary for infection (Ellermann et al. 2020). Enhancing nociceptor activation or endocannabinoid levels pharmacologically may therefore serve as a novel therapeutic strategy against infection with enteric pathogens.

## Post-infectious irritable bowel syndrome

A particularly intriguing example of the link between enteric pathogens and the microbiome-gut-brain axis is the occurrence of sustained and new-onset symptoms of irritable bowel syndrome (IBS) in a subset of patients who recover from an acute bout of infectious diarrhea. This relationship was thoroughly cemented following a significant outbreak of waterborne dysentery in Walkerton, Ontario, caused by contamination of the town’s water supply with *E. coli* O157 (EHEC) and *Campylobacter jejuni*, where at least seven people died and more than 2300 residents were affected. Detailed follow-up of impacted individuals showed that around one-third went on to display persistent symptoms of diarrhea-predominant IBS, with women and girls, those of younger age, and those who had experienced prolonged and bloody diarrhea with cramping most likely to be affected (Marshall et al. 2006). Subsequent work places the prevalence of post-infectious IBS at around 10% of those recovering from infectious gastroenteritis, but this still represents a significant number of individuals. Gut dysfunction may be initiated by epithelial barrier defects, inflammation, and injury to components of the enteric nervous system, and thereafter sustained by a dysbiotic microbiome, persistent cycles of inflammation, and ongoing enteric neural afferent signaling with increased visceral sensitivity and accompanying pain (Downs et al. 2017, Lupu et al. 2023, Taghaddos et al. 2024). The observation of long-standing dysbiosis in the condition has led to the suggestion that microbiome-targeted therapies, such as FMT, may be of benefit, with some emerging evidence of success (Tkach et al. 2022, Taghaddos et al. 2024).

The presence of GI symptoms and viral shedding into feces from patients with SARS-CoV-2 has highlighted the GI tract as a common site of infection by this respiratory viral pathogen in a subset of patients. In particular, the emergence of gut-brain deficits following SARS-CoV-2 infection has implicated respiratory viruses as potentially causing post-infectious IBS in a subset of patients (Gareau and Barrett 2023). A post-hoc analysis of a prospective multicenter study of patients developing post-SARS-CoV-2 symptoms reflecting diseases of gut-brain interaction (DGBI) found an overlap of those symptoms with those seen in patients with IBS (Marasco et al. 2025). Similarly, another respiratory viral pathogen, influenza A, can cause GI symptoms, including diarrhea, vomiting, and abdominal pain, suggesting a non-respiratory route of infection (Bullock et al. 2025). At this time, in contrast to SARS-CoV-2, a gut-brain pathway for influenza A has not yet been described, suggesting perhaps that not all respiratory viral pathogens can impact the MGB axis.

## Microbiome-dependent mechanisms and therapeutics

Therapeutic modification of the gut microbiome and/or replacement of its functions through multiple different means, including probiotics, bacterial metabolites (e.g. SCFA), and dietary manipulation, have shown promising results in ameliorating or limiting colonization and disease following enteric pathogen infection (Fig. 2).

**Figure 2:**
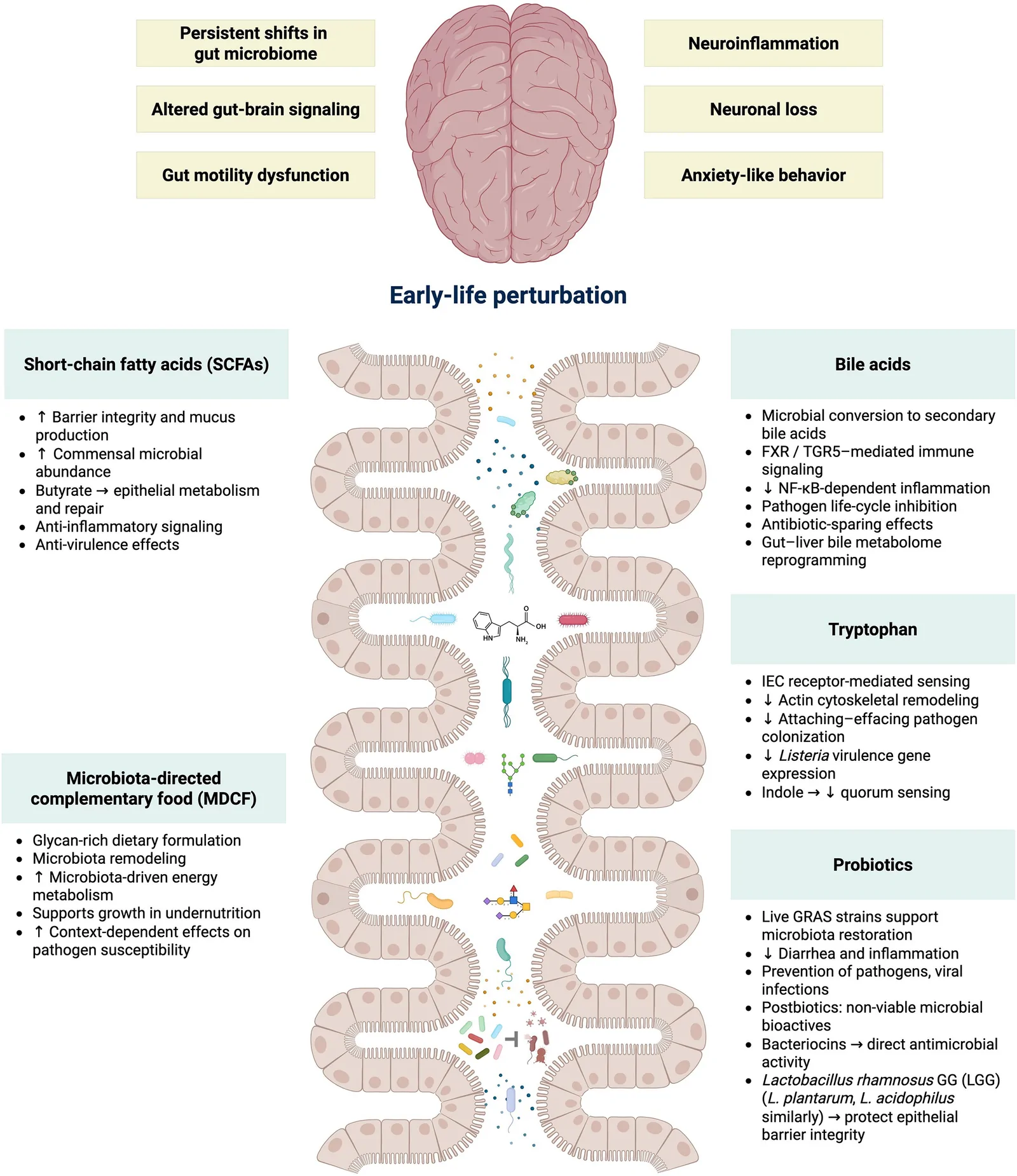
Mechanisms and therapeutic modulation of host–microbe interactions following early-life enteric perturbation. Early-life enteric perturbations, including infection and antibiotic exposure, can induce long-term alterations in gut–brain communication, leading to persistent changes in microbiome composition, gut motility, neuroinflammation, neuronal loss, and anxiety-like behavior. Therapeutic modulation of the gut microbiome or replacement of microbial functions offers a strategy to limit pathogen colonization and disease. Short-chain fatty acids (SCFA), particularly butyrate, promote epithelial repair, mucus secretion, and reduced inflammatory signaling while exerting anti-virulence effects against enteric pathogens. Microbiota-directed complementary food (MDCF) formulations are glycan-rich dietary therapeutics that remodel the gut microbiota and enhance microbial glycan metabolism to support energy balance and growth, with effects that may be context-dependent. Bile acids represent a host–microbe signaling network in which microbial conversion to secondary bile acids regulates pathogen life-cycle processes and innate immune responses via FXR and TGR5 signaling. Tryptophan metabolites modulate epithelial signaling by binding dopamine receptors on intestinal epithelial cells, reducing host actin regulatory proteins required for attaching-and-effacing pathogen colonization and suppressing pathogen virulence. Probiotic and postbiotic interventions restore microbiota composition, reduce diarrhea and inflammation, and enhance epithelial integrity, with postbiotics providing non-viable bioactive molecules that exert direct antimicrobial effects.

**Figure 3:**
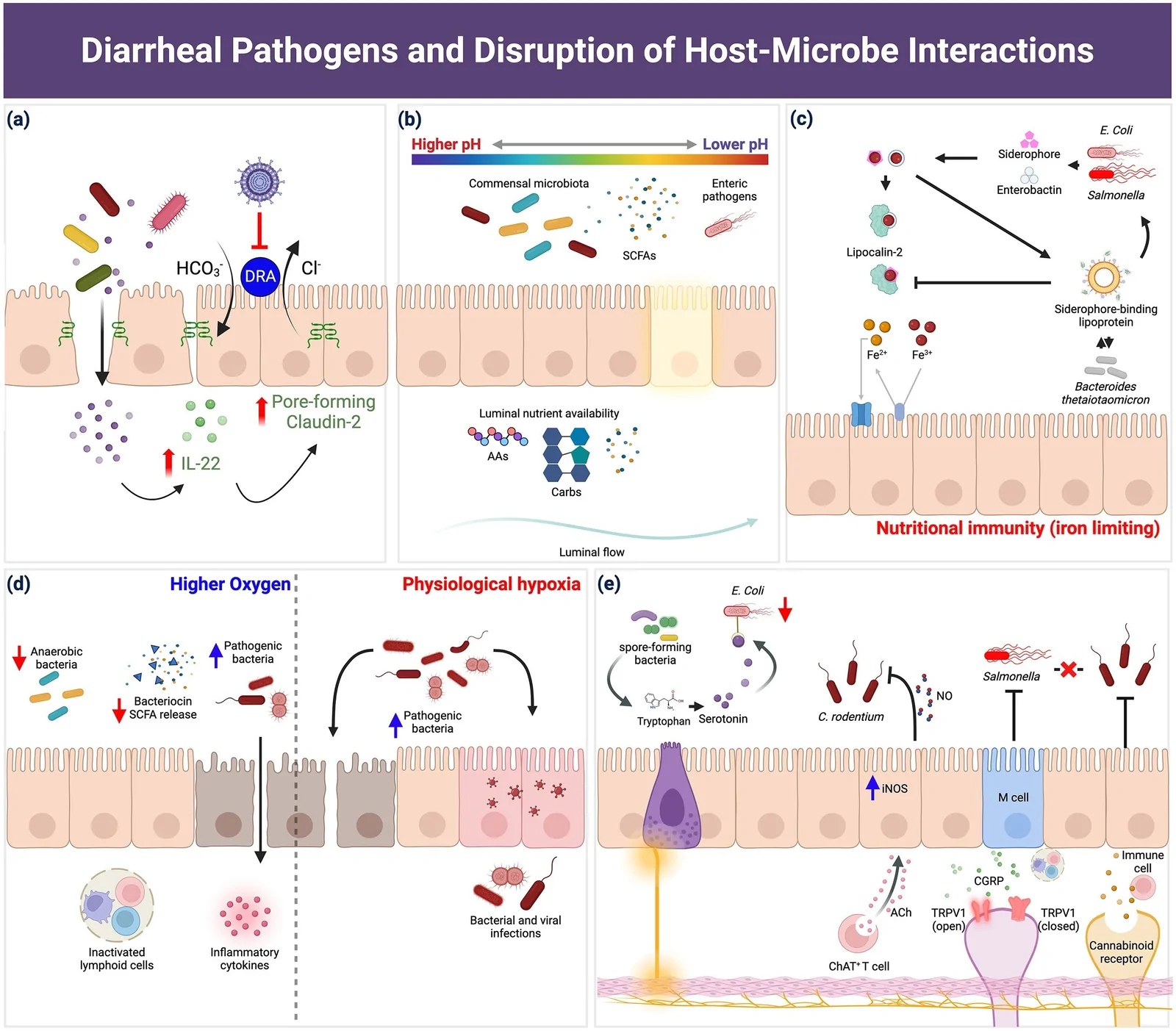
Diarrheal pathogens and disruption of host–microbe interactions. **(a) Host epithelial responses**. Enteric bacterial and viral pathogens disrupt intestinal epithelial cell (IEC) function by inhibiting absorptive solute transporters, including the DRA Cl⁻/HCO₃⁻ exchanger, leading to impaired electrolyte absorption and increased fluid secretion. Pathogen-induced IL-22 promotes expression of the pore-forming tight junction protein Claudin-2, enhancing paracellular water flux (“leak-flux” diarrhea) and contributing to pathogen clearance and diarrheal disease. **(b) Luminal factors**. The intestinal lumen integrates physicochemical and microbial cues that regulate colonization resistance. Luminal pH gradients shape regional pathogen niches, while the commensal microbiota limits enteric pathogens through nutrient competition and metabolite production, including short-chain fatty acids (SCFA). Luminal nutrient availability (carbohydrates and amino acids) and luminal flow further influence pathogen expansion and washout in a diet-dependent manner. **(c) Iron sequestration**. Iron availability in the lumen is controlled by host–microbe competition as part of nutritional immunity. Enteric pathogens such as *E. coli* and Salmonella produce siderophores, including enterobactin, to scavenge Fe²⁺/Fe³⁺ and promote colonization. Host-derived lipocalin-2 and commensal bacteria such as *Bacteroides thetaiotaomicron*, via siderophore-binding lipoproteins, intercept pathogen siderophores and restrict iron acquisition. **(d) Hypoxia**. Oxygen tension varies along the gut, with the colon characterized by physiological hypoxia, shaping microbiome composition and metabolite production. Altered oxygen availability affects anaerobic bacteria, SCFA release, inflammatory cytokine production, and pathogen expansion, influencing susceptibility to bacterial and viral infections. **(e) Neurotransmitters**. Neurotransmitters modulate host–microbe interactions and influence enteric pathogen behavior. Serotonin (5-HT), produced by enteroendocrine cells and modulated by spore-forming bacteria, suppresses virulence gene expression in attaching-and-effacing pathogens (e.g. *E. coli* and *Citrobacter rodentium*) through the bacterial serotonin receptor CpxA. Immune cell–derived acetylcholine (ACh) from ChAT⁺ T cells limits inflammation and disease severity during enteric infection. TRPV1⁺ nociceptor neurons respond to pathogens by releasing calcitonin gene-related peptide (CGRP) via pathways involving M cells, restricting pathogen invasion. In addition, cannabinoids acting through immune cells inhibit pathogen virulence and contribute to host defense against enteric infection.

### SCFA

As noted previously, SCFA levels in the GI tract are often correlated with overall health and are used as a surrogate for colonization by commensal (beneficial) microbes. In addition, SCFA may be used therapeutically to help alleviate disease following enteric infection and/or altered colonization of the GI tract. Following *C. rodentium* infection in mice, for example, rectal administration of butyrate improved disease outcomes, including reversal of weight loss and decreased food intake, as well as inducing tissue repair, reduced inflammation, and improved mucus secretion (Jiminez et al. 2017). Inflammation following infection with *Campylobacter jejuni* in mice was also attenuated by supplementation with the SCFA, butyrate, accompanied by decreased diarrhea and reduced pro-inflammatory cytokines (Du et al. 2022). Lastly, sodium butyrate has been shown to demonstrate anti-virulence properties that limit toxin production by *V. cholerae in vitro* (human IEC and a rabbit intestinal loop model) and *in vivo* (neonatal mice), suggesting it may serve as an alternative or adjunctive therapeutic strategy in cholera infection (Kundu et al. 2025). Supplementation with SCFA has also been shown to improve behavior and cognition in models of gut-brain deficits. For example, neonatal administration of antibiotics to mice disrupted colonization of the GI tract and caused behavioral deficits that could be restored by supplementation with tributyrin (Keogh et al. 2021). In sepsis triggered by cecal ligation and puncture in mice, neuroinflammation and cognitive deficits were also improved by SCFA administration (Zhao et al. 2026).

In a neonatal mouse model of *Cryptosporidium* infection, the gut microbiome was found to produce long-chain unsaturated fatty acids that appeared to enhance parasitic invasion into IEC and may contribute to susceptibility in infants and young children (VanDussen et al. 2020). Interestingly, *Cryptosporidium* infection in mice leads to increased SCFA levels in the context of an increase in abundance of bacteria associated with a stressed gut environment. Because of these seemingly discordant findings, it is not clear whether increases in fatty acid levels are actually beneficial or detrimental (Karpe et al. 2021). It is also important to note that this study was performed in mice at weaning, rather than in neonates, which may impact parasite-microbiome interactions.

### Dietary therapeutics

Microbiota-directed complementary food (MDCF) formulations are novel dietary therapeutics designed specifically for malnourished children (Chen et al. 2021). They were designed using metabolomic and proteomic analyses of plasma and fecal samples of children with severe acute malnutrition (Gehrig et al. 2019). Their make-up is designed to alter the gut microbiome and promote glycan metabolism to improve growth responses (Hibberd et al. 2024). Recent studies have found that certain bacterial species, such as *Prevotella copri*, could utilize MCDF glycans to promote their own growth and modulate energy metabolism in human hosts via a novel structure-function relationship between glycans and microbes (Chang et al. 2024). While this may be beneficial in promoting weight gain in malnourished individuals, an increased *Prevotella* burden may have detrimental impacts, including promoting infection with certain enteric pathogens such as *Listeria* (Rolhion et al. 2019). Moreover, while behavior and cognitive function have not been studied thoroughly during MCDF supplementation, an improvement in neurodevelopment in children with moderate acute malnutrition following supplementation for three months (Mostafa et al. 2024) would suggest that this microbiome-related feeding likely has a beneficial impact on behavior, but this remains to be determined. No matter what, diet remains a powerful method by which the luminal environment can be modified for health benefits and to reduce the risk of enteric infection.

### Bile acids

Bile acids are an important class of metabolites produced by the host (primary bile acids) and subsequently modified by the gut microbiome (secondary bile acids), and which can signal to the innate immune system in both health and disease. Their composition can be manipulated by the diet and exposure to antibiotics, making them a crucial signaling network during enteric infection (Collins et al. 2023). Bacterially derived bile acids were found to be protective in mouse norovirus infection, whereas host-produced conjugated bile acids are proviral. Therefore, the balance of bile acids within the gut lumen can determine host susceptibility to norovirus infection (Peiper et al. 2024). Similarly, in parasitic infection, secondary bile acids can potently inhibit *Cryptosporidium* growth *in vitro* (Funkhouser-Jones et al. 2023), suggesting they may serve a protective role *in vivo*.

Dietary supplementation with deoxycholic acid, a secondary bile acid, also improved survival, reversed growth inhibition, and alleviated GI disease in necrotic enteritis caused by *Clostridium perfringens* in chickens, serving as a promising alternative to antibiotics (Whitmore et al. 2025). In *C. difficile* infection, the secondary bile acid ursodeoxycholic acid (or ursodiol) inhibits the life cycle of multiple strains *in vitro* through dose-dependently inhibiting spore germination, growth, and toxin activity (Winston et al. 2020). *In vivo*, using a mouse infection model, ursodiol pretreatment could attenuate *C. difficile* pathogenesis through the bile acid receptors, farnesoid X receptor (FXR), and transmembrane G-protein coupled membrane receptor 5 (TGR5), which can modulate host innate immune responses via NF-kB signaling, reducing a detrimental pro-inflammatory response in the host (Winston et al. 2020). Metabolomic studies in mice found that enteric infections can relay signals between the gut and liver to induce transcriptional reprogramming to shape the bile metabolome, and subsequently modify the host defense response to infection (Zhang et al. 2024). Taken together, these findings suggest that provision of secondary bile acids may serve as a novel treatment strategy for bacterial infections, in part via the innate immune system. While studies using bile acids to modulate enteric infection-induced behavioral impairments are currently lacking, lower serum concentrations of bile acids were strongly associated with cognitive decline in Alzheimer’s patients, suggesting an important role for bile acids in modulating the MGB axis (Mahmoudian Dehkordi et al. 2019).

### Tryptophan

Tryptophan is an important dietary metabolite known to modulate both GI function and gut-brain communication. Tryptophan metabolites can bind to dopamine receptors on IEC, decreasing the expression of host actin regulatory proteins that are important for colonization of attaching and effacing pathogens, including *C. rodentium* (Scott et al. 2024). Tryptophan derivatives, including serotonin and indole, also serve as cues for enteric pathogen colonization and can interfere with pathogen virulence (Hoskan and Sperandio 2025). Indole, for example, may act as a quorum-sensing molecule, causing diminished biofilm production and decreasing virulence factor production following infection with *Listeria monocytogenes in vitro* (Rattanaphan et al. 2020). In *C. rodentium* infection of mice, long-term alterations in tryptophan metabolism were identified, and post-infectious cognitive defects could be ameliorated by supplementation with an IL22-producing probiotic *Lactococcus lactis* strain, which restored the kynurenine/tryptophan ratio (Meynier et al. 2022).

### Pro- and postbiotics

Probiotic bacteria, strains considered to be “generally regarded as safe” (GRAS) and known to provide a benefit to the host, are commonly used as a safe therapeutic for enteric pathogens. They can help reduce diarrhea, ameliorate inflammation, and restore the gut microbiome post-infection. Given that probiotic treatment requires the administration of live bacterial strains, postbiotics have also gained some attention as an alternative to probiotic bacteria because they are functional bioactive molecules rather than live organisms, reducing the risk of bacteremia in vulnerable populations. They are thought to act via multiple mechanisms, similar to those seen in probiotic supplementation studies, including modification of the gut microbiome, improvement of intestinal epithelial barrier function, and regulation of mucosal immune responses and metabolic pathways (Thakur and Kaur 2025). Postbiotics can contain bacteriocins, exopolysaccharides, and other proteins secreted from probiotic strains that have beneficial biological activities within the host. Thus, a postbiotic preparation from *Lacticaseibacillus rhamnosus* strain GG (LGG) was able to prevent SARS-CoV-2 spike protein-induced secretory and permeability deficits in human colonic epithelial cells *in vitro* (Poeta et al. 2023). Similarly, a postbiotic bacteriocin isolated from *Lactiplantibacillus plantarum* and *Lactobacillus acidophilus* demonstrated strong antimicrobial activity against *E. coli, Salmonella*, and *Staphylococcus* after a 16 h exposure *in vitro* (Rahman et al. 2025). *Citrobacter rodentium*-induced impairments in cognitive function could also be restored by supplementation with probiotic bacteria (both *L. lactis* (Meynier et al. 2022) as well as a *L. rhamnosus/Lactobacillus helveticus* combination (Gareau et al. 2011)), highlighting the ability of probiotics to improve gut-brain impairments caused by enteric pathogens.

### Impact of therapies in elderly subjects

Most of the mechanisms of infection of diarrheal pathogens and proposed treatments have been studied in neonatal and early life patients, or in pre-clinical models. Limited studies exist in elderly populations or models, despite strong clinical associations with worsened outcomes. Elderly patients are highly susceptible to norovirus, *C. difficile, Salmonella*, and parasitic pathogens. Probiotics are also commonly used in elderly patients, often to ameliorate constipation. A recent randomized-placebo controlled trial in middle-aged and older adults with chronic constipation found that multi-strain probiotic supplementation for 28 days improved bowel movement frequency, bloating, and favorably remodeled the gut microbiome, increasing SCFA biosynthesis pathways as determined by metabolomics (Xiao et al. 2026). Supplementation with 5-HT in older adults likewise improved the composition of the gut microbiome after 12 weeks in individuals with poor sleep quality, including an increase in the abundance of SCFA-producers and leading to an increase in alpha diversity (Sutanto et al. 2024). While more studies are needed, particularly in patients suffering from enteric pathogen-associated diarrhea, many of the mechanisms that contribute to altered host-microbe interactions in early life are likely to be important in elderly individuals, and many therapeutic strategies may also be applicable to this population.

## Conclusions and future directions

The restoration of healthy host-microbe interactions following disruption caused by enteric pathogens or other pathways remains an important therapeutic goal. The combination of signaling mechanisms in the gut that influence a healthy state makes this process complex, but at the same time increases the options by which therapies can act. Combination therapies to target multiple pathways represent an exciting proposition, through which pathogens can be more quickly neutralized and homeostasis within the host microbiome more readily restored. The ultimate goal would be to limit not only the local disruption to the microbiome that can occur at various life stages or post-infections, but also the associated derangements in gut-brain signaling and possible long-term sequelae of cognitive dysfunction and disruption of other body systems. While therapeutic interventions likely need to be simple and inexpensive to have a maximal impact in areas of the world where diarrheal diseases remain common, or following natural disasters or catastrophic outbreaks of foodborne infections, continued focus on the mechanisms discussed here should shed light on novel approaches that fulfill these criteria.

## Supplementary Material

lxag237_Supplemental_File

## Data Availability

No data were generated in the context of article preparation, and thus there are no source datasets relevant to this review article.
